# Sirtuins in metabolism, DNA repair and cancer

**DOI:** 10.1186/s13046-016-0461-5

**Published:** 2016-12-05

**Authors:** Zhen Mei, Xian Zhang, Jiarong Yi, Junjie Huang, Jian He, Yongguang Tao

**Affiliations:** 1 Key Laboratory of Carcinogenesis and Cancer Invasion of Ministry of Education, Xiangya Hospital, Central South University, 87 Xiangya Road, Changsha, Hunan 410008 China; 2 Cancer Research Institute, School of Basic Medicine, Central South University, Changsha, Hunan 410078 China

**Keywords:** Sirtuin, DNA damage, Metabolism, Cancer, Post-translation modification

## Abstract

The mammalian sirtuin family has attracted tremendous attention over the past few years as stress adaptors and post-translational modifier. They have involved in diverse cellular processes including DNA repair, energy metabolism, and tumorigenesis. Notably, genomic instability and metabolic reprogramming are two of characteristic hallmarks in cancer. In this review, we summarize current knowledge on the functions of sirtuins mainly regarding DNA repair and energy metabolism, and further discuss the implication of sirtuins in cancer specifically by regulating genome integrity and cancer-related metabolism.

## Background

**Fig. 1:**
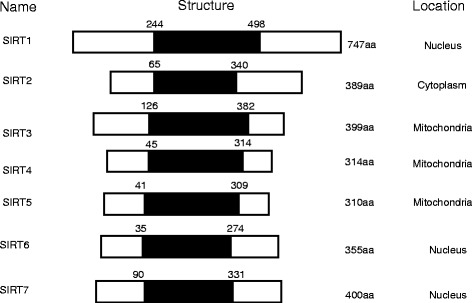
Schematic representation of seven mammalian sirtuins. The shaded area represents NAD^+^ - dependent catalytic domain. aa, amino acids

Sirtuins, the highly conserved NAD + −dependent enzymes, are mammalian homologs of the yeast Sir2 gene which has been known to promote replicative life span and mediate gene silencing in yeast [1]. The sirtuin family comprises seven proteins denoted as SIRT1-SIRT7, which share a highly conserved NAD + −binding catalytic domain but vary in N and C-termini (Fig. 1). The divergent terminal extensions account for their various subcellular localization, enzymatic activity and binding targets. SIRT1, SIRT6, and SIRT7, are chiefly nuclear proteins, while SIRT3, SIRT4 and SIRT5 predominantly reside in mitochondria and SIRT2 is primarily cytosolic (Fig. 1). But some of theses proteins are reported to translocate from their typical compartments under specific circumstances [2–4]. Besides the well-recognized deacetylase function, sirtuins have also evolved as mono ADP ribosyltransferase, lipoamidase (SIRT4), demalonylase and desuccinylase (SIRT5) [5, 6].

The host cells are constantly subjected to oxidative, genotoxic and metabolic stress. The ratio of NAD+/NADH is correlated with stress resistance, oxidative metabolism and DNA repair [7]. Sensing intracellular NAD+ changes, sirtuins are proposed to work as stress adaptors. Meanwhile, given their diverse enzymatic activities, they are described to play critical roles in regulating post-translational modifications (PTMs), among which acetylation is an important form. Sirtuins deacetylate a multitude of targets including histones, transcription factors, and metabolic enzymes. Taken together, sirtuins have been implicated in numerous cellular processes including stress response, DNA repair, energy metabolism, and tumorigenesis [8, 9].

Aberrant cellular metabolism in cancer cells characterized by elevated aerobic glycolysis and extensive glutaminolysis [10] is essential to fuel uncontrolled proliferation and malignant tumor growth. The Warburg effect, which describes that tumor cells preferentially use glucose for aerobic glycolysis in the presence of ample oxygen [11], has emerged as one of hallmarks of cancer. Even though originally thought to be energy insufficient, Warburg effect is now widely accepted to confer rapid proliferation and invasive properties to tumor cells [12–14]. In parallel, many cancer cells exhibits enhanced glutamine metabolism and cannot survive in the absence of glutamine [15]. Recent studies have shown that a succession of well-established oncogenic cues, including Myc, Ras or mammalian target of rapamycin complex 1 (mTORC1) pathways play imperative roles in inducing glutaminolysis [16–18]. Besides metabolic reprogramming, deregulated DNA-repair pathways and subsequent genome instability appears to facilitate the acquisition of tumorigenic mutations propitious to tumor growth and cancer progression [19, 20].

Mounting evidence has shed light on that sirtuins play diverse parts in cancer [1]. In this review, we summarize an overview and update on the function of sirtuins in metabolism and DNA repair, and further touch on their roles in cancer mainly by affecting genome integrity and cancer-associated metabolism.

## Sirtuins in metabolism

### Glucose metabolism

**Fig. 2:**
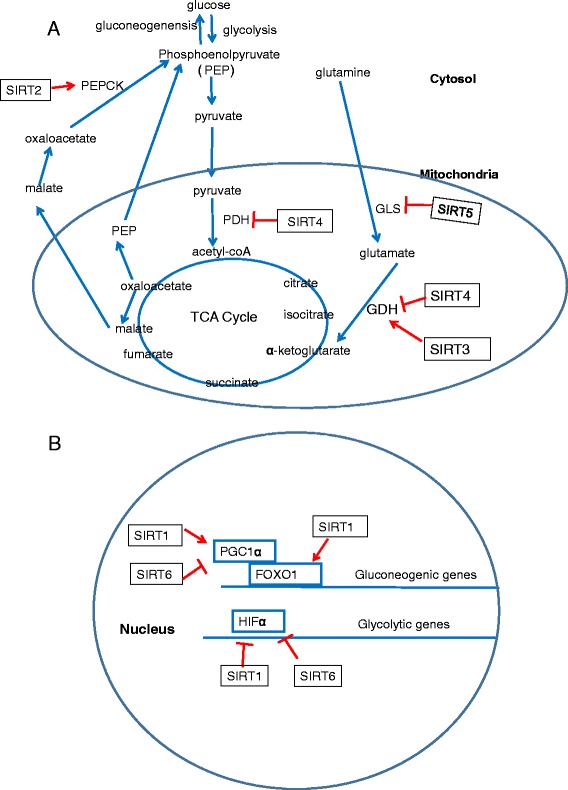
Overview of sirtuins in glucose metabolism. Selected pathways in nucleus, cytosol and mitochondria are depicted. **a** Located in cytoplasm, SIRT2 deacetylates the rate-limiting enzyme PEPCK and promotes gluconeogenesis during low nutrient condition. Both SIRT3 and SIRT4 target GDH in mitochondria but their enzymatic activities seem to be opposite. Besides GDH, SIRT4 also reduces PDH activity which converts pyruvate to acetyl CoA. SIRT5 facilitates glycolysis via glycolytic enzyme GAPDH and may disrupt glutamine metabolism through GLS. **b** In respect to the nuclear sirtuins, both SIRT1 and SIRT6 suppress the transcription factor HIF1α through different manners and subsequently attenuate glycolysis. The reciprocal activation of FOXO1 and its coactivator PGC-1α by SIRT1 reinforces the gluconeogenic transcription. By contrast, SIRT6 down-regulates PGC-1α and suppresses hepatic glucose production. PEPCK,phosphoenolpyruvate carboxykinase; GDH,glutamate dehydrogenase; PDH,pyruvate dehydrogenase; GAPDH,glyceraldehyde phosphate dehydrogenase; GLS,glutaminase; PGC-1α,Peroxisome proliferator-activated receptor gamma coactivator 1 α; FOXO1,forkhead box protein O1

Glucose metabolism encompasses several processes implicating glucose uptake, utilization, storage and output, which needs elaborate coordination among the regulating hormone insulin and its counterpart such as glucagon. Sirtuins are verified to exert various impacts on gluconeogenesis, glycolysis, insulin secretion and sensitivity bearing therapeutic potential to several metabolic diseases (Fig. 2).

#### SIRT1

SIRT1 is the most conserved mammalian NAD + −dependent protein deacetylase that has emerged as a regulator of glucose metabolism. As for gluconeogenesis, the role of SIRT1 is regarded as dual and intricate. In a short-term fasting phase, SIRT1 induces decreased hepatic glucose production by suppressing CRTC2 (CREB-regulated transcription coactivator 2), a key mediator of early phase gluconeogenesis [21]. With the fasting phase prolonged, SIRT1 deacetylates and activates both the transcription factor FOXO1 (forkhead box protein O1) and its co-activator PGC1α (Peroxisome proliferator-activated receptor gamma coactivator 1 α) [22, 23], which reinforces the gluconeogenic transcriptional program. In respect to glycolysis, SIRT1 attenuates the transcription of glycolytic genes by directly deacetylating transcription factor HIF1α [24] and also inhibits glycolytic enzyme PGAM1 (phosphoglycerate mutase 1) through deactylation [25]. SIRT1 is also implicated in glucose metabolism by functioning as an insulin sensitizer. Through transcriptionally repressing the uncoupling protein 2 (UCP2), SIRT1 positively modulates glucose-stimulated insulin secretion [26]. Accumulating evidence suggests that SIRT1 and SIRT1 activators can prevent and reverse insulin resistance and diabetic complications, proven to be a promising therapeutic target in type 2 diabetes (T2D) [27–30].

#### SIRT2

Compared to SIRT1, SIRT2 is predominantly a cytoplasmic protein and pretty abundant in adipocytes. SIRT2 activates the rate-limiting enzyme phosphoenolpyruvate carboxykinase (PEPCK) via deacetylation and enhances gluconeogenesis during times of glucose deprivation [31]. Meanwhile recent studies have proposed that, in regard to insulin sensitivity, SIRT2 may act specific and opposing roles in different tissues [32].

#### SIRT3, SIRT4, and SIRT5

Primarily located in mitochondria, SIRT3, SIRT4, and SIRT5 sense and regulate the energy status in this organelle. Activating glutamate dehydrogenase (GDH), SIRT3 facilitates gluconeogenesis from amino acids [33]. In addition, SIRT3 indirectly destabilizes transcription factor HIF1α and subsequently inhibits glycolysis and glucose oxidation [34]. Intriguingly, recent studies have shown that SIRT3 levels in pancreatic islets are reduced in patients afflicted with type 2 diabetes [35] and SIRT3 overexpression in pancreatic β-cells promotes insulin secretion and abrogates endoplasmic reticulum (ER) stress that is connected to β-cell dysfunction and apoptosis [36].

SIRT4, initially reported as a unique ADP-ribosyltransferase, appears to blunt insulin secretion by reducing GDH activity [37]. In line with this, the amino acid-stimulated insulin secretion is upregulated in SIRT4-deficient insulinoma cells [38]. Besides GDH, a diverse range of SIRT4 targets are identified in the regulation of insulin secretion including ADP/ATP carrier proteins, insulin-degrading enzyme, ANT2 and ANT3 [37]. Interestingly SIRT4 is characterized as a lipoamidase and diminishes pyruvate dehydrogenase complex (PDH) activity, an enzyme converting pyruvate to acetyl CoA and connecting glycolysis to the TCA cycle [6].

In contrast to other sirtuins, SIRT5 displays deacetylase and NAD + −dependent demalonylase and desuccinylase activities. SIRT5 facilitates glycolysis via demalonylating the glycolytic enzyme glyceraldehyde phosphate dehydrogenase (GAPDH) [39]. And a recent study proposed that SIRT5 might be positively correlated with insulin sensitivity, the biological significance of which still remains to be confirmed [40].

#### SIRT6

There is growing appreciation that SIRT6 plays a critical role in glucose homeostasis. In the case of gluconeogenesis, SIRT6 indirectly suppresses PGC-1α leading to downregulation of hepatic glucose production [41]. Similar to SIRT1, SIRT6 can also shut down the glycolytic flux by deacetylation of histone H3 lysine 9 (H3K9) in promoters of glycolytic genes and acting as a HIF1α corepressor [42]. In this regard, one recent study revealed that the antiglycolytic activity of SIRT6 exerts beneficial impact against nasal polyp formation [43]. Meanwhile, SIRT6 may positively mediate glucose-stimulated insulin secretion [44] and overexpression of it enhances insulin sensitivity in skeletal muscle and liver, emerging as an attractive therapeutic target for T2D.

#### SIRT7

SIRT1, SIRT3 and SIRT6, as discussed above, have all been identified to exert repressive effect on HIF-1 activity through different mechanisms. Likewise, it was reported that Sirt7 overexpression decreased HIF-1α and HIF-2α protein levels through a distinct mechanism independent of its deacetylase activity [45]. Besides, Sirt7 knockout mice were resistant to glucose intolerance and insulin sensitivity is improved in Sirt7 knockout mice receiving a high-fat diet [46]. All these findings revealed a novel role for SIRT7 in glucose metabolism.

### Lipid metabolism

**Fig. 3:**
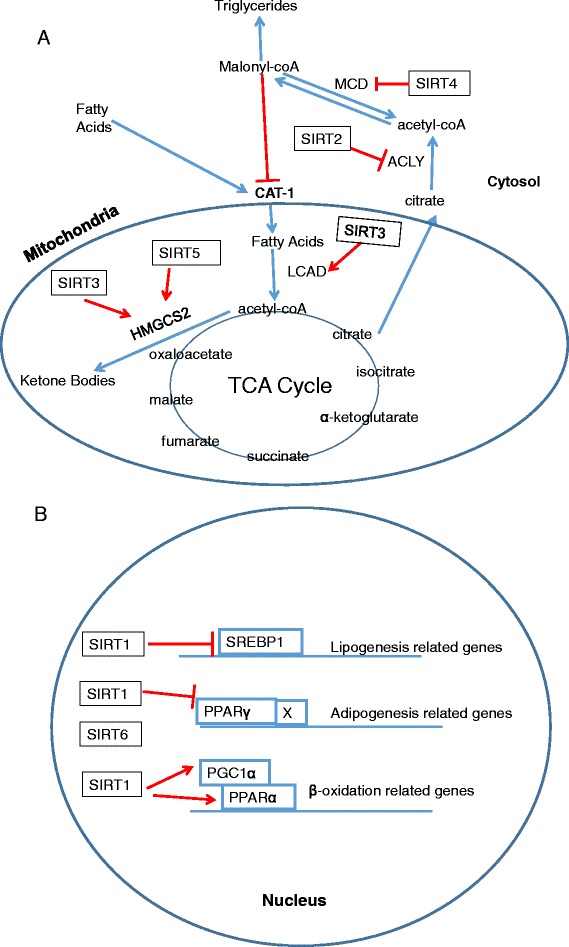
Overview of sirtuins in lipid metabolism. Selected pathways in nucleus, cytosol and mitochondria are depicted. **a** Activating LCAD, a key enzyme in long-chain fatty acids oxidation, SIRT3 increases β-oxidation in hepatocytes and skeletal muscle. Both SIRT3 and SIRT5 promotes ketogenesis via HMGCS2 in liver. In cytoplasm, SIRT2 deacetylates ACLY and deters lipid synthesis. In contrast to SIRT3, SIRT4 inhibits MCD and contributes to increased malonyl CoA,which suppresses the fatty acid translocator CAT-1 and shuts down entery of fatty acid for β-oxidation. **b** SIRT1 and SIRT6 reduce the activity of nuclear hormone receptor PPARγand lead to decreased adipogenesis. SIRT1 also destabilizes SREBP1 and transcriptionally represses lipogenesis. Besides the negative regulation, SIRT1 boosts fatty acid oxidation by enhancing PPARα and its coactivator PGC1α. LCAD, long chain acyl CoA dehydrogenase; HMGCS2,3-hydroxy-3-methylglutaryl CoA synthase 2; ACLY,ATP citrate lyase; MCD,malonyl CoA decarboxylase; CAT-1,carnitine acyl transferase-1; SREBP1,sterol regulatory element binding protein 1

Lipid homeostasis is maintained by a collection of metabolic pathways including hepatic lipogenesis, adipogenesis, lipolysis in white adipose tissue (WAT) and lipid utilization in both liver and skeletal muscle, each of which is dominant under distinct nutrient condition. Sirtuins are involved in multiple aspects of lipid metabolism and related diseases as briefly summarized below (Fig. 3).

#### SIRT1

In addition to its critical roles in glucose metabolism, SIRT1 is also convinced to regulate lipid homeostasis. During fasting, SIRT1 deacetylates and destabilizes sterol regulatory element binding protein 1 (SREBP1), a hepatic transcription factors for lipogenesis and cholesterol synthesis, which theoretically represses fatty acid and cholesterol synthesis [47]. In agreement with this, a recent study points out an increase of de novo lipogenesis upon SIRT1 inhibition in human fetal hepatocytes [48]. Moreover, the complex transcriptional program controlling adipogenesis is mainly coordinated by the nuclear receptor PPARγ. SIRT1 reduces the activity of PPARγ and suppresses adipogenesis in situation of nutrient depletion, subsequently engendering increased lipolysis and fat mobilization from WAT [49].

SIRT1 also occupies a special place in lipid metabolism via enhancing lipid oxidation. In support of this notion, activation of PPARα and its coactivator PGC1α by SIRT1 are presented in both liver and skeletal muscle, which stimulates the expression of β-oxidation related genes [50, 51]. Of note, AMPK, an energy sensor, is also involved in fatty acid oxidation in skeletal muscle and its interaction with SIRT1 is described as a reciprocal regulatory loop, in which AMPK upregulates SIRT1 by boosting NAD+ levels and SIRT1 induces the AMPK activator [52, 53]. Given its diverse functions in lipid homeostasis, SIRT1 is a potential therapeutic target to prevent obesity and liver steatosis and ameliorate cardiovascular diseases in obese individuals [54–56].

#### SIRT2

Similarly to SIRT1, SIRT2 facilitates lipolysis in WAT under nutrient deprivation by repressing transcriptional activity of PPARγ [57], and it attenuates lipid synthesis by suppressing ATP citrate lyase (ACLY), a building block for hepatic de novo lipogenesis [58], which might be biologically significant to fatty liver treatment. The link between SIRT2 and fatty acid oxidation appears to be elusive, and the development of diet-induced obesity in mice may be attributed to SIRT2 repression and attendant reduced β-oxidation [59].

#### SIRT3, SIRT4, and SIRT5

Current studies mainly indicate the role of SIRT3 in fatty acid oxidation. SIRT3 activates long chain acyl CoA dehydrogenase (LCAD) [60], a key enzyme involved in long-chain fatty acids oxidation, triggering β-oxidation in hepatocytes and skeletal muscle and also promotes ketogenesis via deacetylating and increasing the activity of the 3-hydroxy-3-methylglutaryl CoA synthase 2 (HMGCS2) in the liver [61].

In contrast to SIRT3, SIRT4 exhibits a negative regulatory role towards fatty acid oxidation. Under nutrient-replete condition, SIRT4 inhibits malonyl CoA decarboxylase (MCD) in muscle, an enzyme converting malonyl CoA to acetyl CoA, favoring lipid anabolism [62]. In parallel, SIRT4 decreases PPARα and its target genes activities, consequently repressing fatty acid oxidation in liver [63]. A more recent study highlighted a novel mechanism that deacetylating and destabilizing MTPα, a key enzyme in β-oxidation by SIRT4 may contribute to the pathogenesis of Nonalcoholic fatty liver disease (NAFLD) [64].

As a desuccinylase described above, SIRT5-dependent desuccinylation and activation of the rate-limiting ketogenic enzyme HMGCS2 may preferentially stimulate ketogenesis and reduced fatty acid oxidation was found in SIRT5 knockout mice [65]. Consistently, downregulated expression of SIRT5 is detected in the liver of NAFLD patients [66].

#### SIRT6

Growing data noted that SIRT6 regulates fat metabolism as well. In the control of lipid storage, SIRT6 reduces the expression of PPARγ dependent genes in adipocytes and overexpression of SIRT6 reverses the detrimental outcome induced by high-fat diet in mice [67]. Additionally, fatty liver and decreased fatty acid oxidation can be seen in liver-specific SIRT6 deletion mice [68], which implies its positive function upon lipogenesis and hepatic β-oxidation. A compelling study even presented that SIRT6 is associated with cholesterol homeostasis by negatively influencing lipogenic transcription factors SREBP1 and SREBP2 [69].

#### SIRT7

SIRT7, regarded as a deacetylase, is hypothesized to link lipid metabolism, even though the recent findings are perplexing and contradictory. The result of Shin et al. indicated Sirt7 knockout mice would develop liver steatosis due to deregulated ER stress [70], while Yoshizawa and colleagues concluded SIRT7-deficient mice were resistant to fatty liver and SIRT7 increases lipogenesis and fat accumulation [46]. Accordingly, the underlying mechanism clearly merits further study.

### Glutamine metabolism

Compared to quiescent cells, proliferating cells prefer to use crucial intermediates derived from tricarboxylic acid (TCA) cycle for biomass building that supports the cell growth and division. Thus, a process called anaplerosis is required to compensate the TCA cycle intermediates, for which glutamine is the main source. In particular, carbon from glutamine contributes to amino acid and fatty acid synthesis while the nitrogen from glutamine is used for nucleotide biosynthesis. During this replenishment, glutamine is firstly converted into glutamate by glutaminase that exists in two versions in mammals, kidney-type glutaminase (GLS) and liver-type glutaminase (GLS2). Glutamate is further converted to TCA cycle intermediates α-ketoglutarate either by glutamate dehydrogenase (GDH) or aminotransferases [71].

A succession of oncogenotypes instigate the upregulated glutamine metabolism [16–18]. The MYC may be the most common oncogene associated with glutamine metabolic rewiring. The oncogenic transcription factor c-Myc, which is known to stimulate cell proliferation through miRNAs regulation, was found to transcriptionally repress miR-23a and miR-23b resulting in a greater expression of their target protein GLS [72] and an enhanced glutamine-fuelled anaplerosis.

Apart from affected by many oncogenic mutations, glutamine enzymes are also controlled through post-translational modifications. SIRT3 might be associated with glutamine metabolism by augmenting the activities of GDH [33] and GLS2 [73] through deacetylation. SIRT4, as mentioned above, inhibits GDH activity by ADP-ribosylation and consequently mediates reduction in glutamine metabolism, which appears to be tumor suppressive as discussed below. Interestingly, SIRT5 might be involved in glutamine metabolism by inhibitory desuccinylating GLS [74].

Clearly, the nuclear sirtuins possess a special place in glucose and lipid metabolism by enhancing or compromising the specific transcriptional program, while SIRT2 and mitochondrial sirtuins mainly aim at metabolic enzymes in response to various nutrient conditions. Most sirtuins are generally expected to promote catabolism such as gluconeogenesis and lipid oxidation and counteract anabolism including glycolysis, lipogenesis, adipogenesis and glutaminolysis. Consistent with this hypothesis, they are potentially involved in treatment of several metabolic diseases and even perhaps MYC-driven tumors (mitochondrial sirtuins).

### Sirtuins in DNA repair

**Fig. 4:**
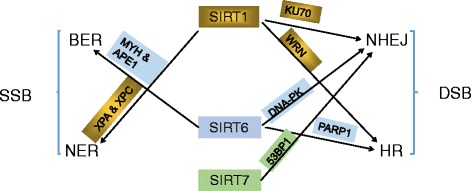
Nuclear sirtuins regulate genomic stability and their roles in the DDR are summarized. SIRT1 is implicated in diverse DNA repair pathways. SIRT1 promotes HR DNA repair by deacetylating WRN, a DNA helicase. It also regulates NHEJ and NER through Ku70 and XPA and XPC after genotoxic stimuli. Like SIRT1, SIRT6 modulates DNA repair pathways at multiple layers. SIRT6 affects BER in a PARP1-depdendent manner and recruits DNA-PK to promote NHEJ. It interacts with two major BER enzymes MYH and APE1 as well. Most recent study uncovered SIRT7 induce NHEJ by recruiting repair factor 53BP1. XPA and XPC, xeroderma pigmentosum A and C; APE1,Apurinic/apyrimidinic endonuclease 1; DNA-PK,DNA-dependent protein kinase

The cells are constantly exposed to genomic insults caused by normal cellular processes or genotoxic agents such as ultra-violet (UV) and ionizing radiation (IR). To fight against genomic instability, eukaryotic cells have developed four major DNA damage response (DDR) pathways, including base-excision repair (BER), nucleotide-excision repair (NER), homologous recombination (HR) and non-homologous end joining (NHEJ) [75]. BER and NER are two repair pathways preferentially for single-strand breaks (SSB) and repair the nucleotides by using the template sister strand. In contrast, for double-strand breaks (DSB), cells are prone to choose either HR or NHEJ. In HR a homologous DNA region from a sister chromatid is used as a template to reconstitute the damaged area [76], while NHEJ modifies and ligates the broken DNA ends with little homology [77]. As explained below, sirtuins have regulated multiple DNA repair pathways and efficiently maintained genomic stability (Fig. 4).

#### SIRT1

Recent studies have highlighted a unique feature of SIRT1 in regulating DNA damage repair as well as its role in maintaining telomere length and genomic stability [78–80]. Upon genotoxic stress, SIRT1 moves from silent promoters to sites of DNA damage, deacetylating histones H1 (Lys26) and H4 (Lys16) and contributing to the recruitment of DNA damage factors [81–84]. SIRT1 is recruited to DSBs in an ATM kinase-dependent manner [85]. This recruitment is important for r-H2AX foci formation and accumulation of the DDR-related proteins such as Rad51, NBS1 and BRCA1 at the breaks.

Important role for SIRT1 in DNA damage repair includes DSB repair by HR [81, 86–88]. SIRT1 promotes HR by deacetylating WRN, a member of the RecQ DNA helicase family with functions in maintenance of genomic stability. Another studies have reported that SIRT1 interacts with telomere in vivo and SIRT1 overexpressed mice display increased HR DNA repair throughout the entire genome [85]. Moreover, SIRT1 is also involved in NHEJ DNA repair. Deacetylation of Ku70 by SIRT1 enhances Ku70-dependent DNA repair and inhibits mitochondrial apoptosis after genotoxic stimuli. SIRT1-dependent KAP1 deacetylation also positively regulates NHEJ [89]. Results establish the functional significance of KAP1 deacetylation in the DDR, highlighting a potential SIRT1-KAP1 regulatory mechanism for DSB repair that is independent from modulating the infrastructure of the chromatin. Finally, SIRT1 can regulate NER by deacetylating and activating xeroderma pigmentosum A and C proteins (XPA and XPC) upon UV damage. Deacetylated XPA and XPC recognize DNA SSBs and recruit other NER factors at the breaks for DNA repair [85].

Also, hMOF plays an important role in DDR, cell cycle progression, and cell growth [90, 91]. hMOF and TIP60, are SIRT1 substrates. The deacetylation of the enzymatic domains of hMOF and TIP60 by SIRT1 inhibits their acetyltransferase activity and promotes ubiquitination-dependent degradation of these proteins. Immediately following DNA damage, the binding of SIRT1 to hMOF and TIP60 is transiently interrupted, with corresponding hMOF/TIP60 hyperacetylation. Lysine-to-arginine mutations in SIRT1-targeted lysines on hMOF and TIP60 repress DNA DSB repair and inhibit the ability of hMOF/TIP60 to induce apoptosis in response to DNA DSB. Together, these findings uncover novel pathways in which SIRT1 dynamically interacts with and provide additional mechanistic insights by which SIRT1 regulates DDR.

#### SIRT2

SIRT2 is initially illustrated to be implicated in mitotic progression and function as a cell cycle regulator [92]. Recently several studies highlighted the critical roles of SIRT2 in genome stability and DDR. During mitosis SIRT2 maintains genome integrity by deacetylating CDH1 and CDC20, co-activators of anaphase promoting complex/cyclosome (APC/C), and regulating APC/C activity [93]. Through the deacetylation of H4K16Ac and the histone methyltransferase (HMT) PR-Set7, SIRT2 modulates H4K20 monomethylation deposition that is paramount in genome stability and DNA repair [94]. Most recently, a study revealed SIRT2 is essential for the ataxia telangiectasia-mutated and Rad3-related (ATR) kinase checkpoint pathway (a pathway maintains genome integrity) and deacetylates CDK9 and ATR-interacting protein (ATRIP) in response to replication stress [95, 96].

#### SIRT3 and SIRT4

SIRT3, SIRT4 and SIRT5, as mentioned above, reside in the mitochondria, where they control numerous aspects of mitochondrial metabolism. Beyond metabolic targets, SIRT3 has been shown to regulate the production of reactive oxygen species (ROS) from mitochondria by multiple mechanisms. For example, SIRT3 deacetylates and activates isocitrate dehydrogenase 2 (IDH2) and manganese superoxide dismutase (MnSOD) [97], which maintains cellular ROS homeostasis. SIRT3 also deacetylates numerous components of the electron transport chain, suggesting that SIRT3 could directly suppress ROS production [98]. In this regard, SIRT3 loss increases cellular ROS levels, contributing to genomic and mitochondrial DNA instability, and SIRT3 KO mice develop estrogen receptor and progesterone receptor-positive mammary tumors [99].

The roles and importance of the mtDNA repair mechanisms and pathways in the protection against carcinogenesis, aging, and other human diseases have been increasingly recognized in the past decade. SIRT3 impacts the repair of mtDNA through its ability to deacetylate OGG1, a DNA glycosylase important in BER, and that loss of SIRT3 results in increases of acetylation, degradation of OGG1 and a decrease of the incision activity of this enzyme and promotes stress-induced apoptosis [100].

Importantly, SIRT3 can bind and deacetylate Ku70 in response to DNA damage [101], suggesting that SIRT3 might be involved in Ku70-dependent DNA repair.

SIRT4 is the most highly induced sirtuins in response to DNA damage stimuli and represses glutamine consumption without affecting glucose uptake, resulting in a decrease in the incorporation of glutamine into the tricarboxylic acid (TCA) cycle intermediates. This metabolic response contributes to cell cycle arrest of damaged cells and promotes the repair of damaged DNA. Indeed, loss of SIRT4 impaired DNA damage-induced cell cycle arrest and resulted in accumulation of DNA damage, and SIRT4 KO MEFs possessed more aneuploidy and exhibited an increased genomic instability.

#### SIRT6

SIRT6, a NAD + −dependent deacetylase and ADP-ribosyltransferase, plays a critical role in numerous DNA repair pathways. The first clues to the function of SIRT6 came from SIRT6 knockout mice. These SIRT6-deficient mice develop striking degenerative phenotypes and dramatic metabolic defects, some of which overlap with pathologies observed in premature aging [102]. SIRT6 was shown to mediate histone (H3K9, H3K56) deacetylation and ultimately maintain the integrity of telomeric chromatin, defects of which likely accounts for the aging like phenotype [103, 104]. At the cellular level, SIRT6 deficiency leads to genomic instability and hypersensitivity to certain forms of genotoxic damage, suggesting its important role in DDR [105].

SIRT6 was initially proposed to work on BER, because the DNA damage sensitivities of SIRT6-deficient cells could be rescued by over-expression of the rate-limiting enzymes in BER [102]. But the definitive role for SIRT6 in BER remains poorly understood. Recently Xu et al. has reported that SIRT6 regulates BER in a PARP1-depdendent manner [106]. H wang et al. further proved that SIRT6 interacts with and stimulates two major BER enzymes (MYH and APE1) and also interacts with the Rad9–Rad1–Hus1 checkpoint clamp which stimulates almost every enzyme in the BER [107].

Besides BER, SIRT6 is also involved in DNA DSB repair such as HR and NHEJ. McCord et al. demonstrated that SIRT6 recruits and stabilizes DNA-dependent protein kinase (DNA-PK) to DSBs in turn promoting NHEJ repair [108]. SIRT6 also stimulates the poly-ADP-ribosyltransferase activity of PARP1 (a protein involved in both double-strand break repair and BER), enhancing NHEJ and HR DNA repair [109]. In addition, the chromatin remodeling factor SNF2H is recruited to DSBs by SIRT6, which provides proper docking sites for downstream DDR factors and allow efficient DNA repair [110]. Taken together, SIRT6 modulates DNA repair pathways at multiple layers.

#### SIRT7

The role of SIRT7 in regulating DNA damage remains largely dormant for years until recently Vazquez and colleagues uncovered a novel function of SIRT7 in DNA repair [111]. They noted that genome homeostasis is disrupted in the absence of SIRT7 and SIRT7 promotes DNA repair by deacetylating lysine 18 of histoneH3 (H3K18Ac) at DNA damage sites and then recruiting NHEJ repair factor 53BP1.

To conclude, SIRT1 and SIRT6 both pave the way for DNA repair partly through histone deacetylation at DNA break sites and then triggering recruitment of multiple repair factors. Besides the histone modifications, they also directly modulate non-histone substrates including DNA repair enzymes and other repair factors. When it comes to mitochondrial sirtuins, things turn out to be more intriguing. SIRT3 affects the genome and mitochondrial DNA stability by maintaining ROS homeostasis and even participate in the mtDNA repair pathways. And SIRT4 is appreciated to ensure proper DNA repair by dampening glutamine metabolism and initiating cell cycle arrest.

### Sirtuins in cancer

It’s now widely believed that sirtuins regulate numerous processes that appear to be awry in cancer cells. The function of sirtuins are characterized as tumor suppressor and/or oncogene, depending on various genetic context, tumor type and stage. As detailed below, we mainly focus on the regulatory roles of sirtuins regarding DNA repair and cancer-related metabolism.

#### SIRT1

SIRT1’s role in carcinogenesis appears to be opposing and complicated. On one hand, SIRT1 was found to be tumorigenic in various human cancer [112–117], which is consistent with its anti-apoptotic activities via p53 and FOXO transcription factors in response to stress [118]. In parallel, SIRT1 was shown to exert an essential role toward the oncogenic signaling mediated by the estrogen receptor-α (ERα) in breast cancer cells [119].

On the other hand, a collection of in vivo mouse models provided evidence that SIRT1 maintains genetic stability in normal cells and decelerates tumor formation [81, 115, 120]. Indeed, a decreased SIRT1 level in breast cancer is associated with BRCA1 mutations, suggesting it as a tumor suppressor. This anti-cancerogenic role could be explained by SIRT1-mediated suppression of the antiapoptotic gene survivin and it is also compatible with SIRT1’s genome stabilizing functions [121]. Interestingly, SIRT1 turned out to be transcriptionally up-regulated by BRCA1, which is best known for its central role as a surveillance factor in DSB repair [122].

Accordingly, this context-dependent role of SIRT1 represents a target for selective killing of cancer versus non-cancer cells [123], and a recent drug screening approach has led to the identification of a potent SIRT1/2 inhibitory substance with potential use in cancer therapy [124].

#### SIRT2

Given the fact SIRT2 maintains genomic stability as discussed above, this sirtuin mainly functions as a tumor suppressor. Notably, it has been revealed that SIRT2 is also linked with cancer metabolism and promotes tumor growth. SIRT2 can regulate the activities of HIF-1α, phosphoglycerate mutase (PGAM) and glucose-6-phosphate dehydrogenase (G6PD) [125–127], which either stimulates glycolytic energy production or coordinates glycolysis and biomass production.

Decreased expression of SIRT2 can be observed in glioma, liver cancer, and esophageal and gastric adenocarcinomas [128, 129]. By contrast, SIRT2 has negative implications in certain types of cancer including acute myeloid leukemia [130], pancreatic cancer, neuroblastoma [131], high-grade human HCC and prostate cancer [132, 133]. Interestingly, SIRT2 functions as both tumor suppressor and oncogene dependent on different tumor grade in breast cancer [134].

#### SIRT3

SIRT3 appears to be a tumor suppressor mainly through its ability to repress reactive oxygen species (ROS) and HIF-1α [34, 135], which fights against metabolic switch towards aerobic glycolysis. In line with this, up-regulation of SIRT3 inhibited the cell growth of oral squamous cell carcinoma (OSCCs) and decreased the levels of basal reactive oxygen species (ROS) in both OSCC lines [136]. Furthermore, a recent study revealed that SIRT3 negatively regulates pancreatic tumor growth by restraining malate-aspartate NADH shuttle, which is critical to sustain glycolysis in tumor cells, via deacetylating glutamate oxaloacetate transaminase (GOT2) [137]. However, in specific type of cancer, SIRT3 turns out to be an oncogene and promote tumorigenensis [138, 139].

#### SIRT4

SIRT4 mRNA level was reduced in several human cancers, such as small cell lung carcinoma [140], gastric cancer [141], breast cancer and leukemia [142]. And lower SIRT4 expression is associated with shorter survival time in lung tumor patients [143]. A recent study has also showed that SIRT4 is a crucial regulator of the stress resistance in cancer cells and SIRT4 loss sensitizes cells to DNA damage or ER stress [144]. Indeed, the activation of mammalian target of rapamycin complex 1 (mTORC1) has been demonstrated to be associated with increased glutamine metabolism through mechanically inhibiting SIRT4 [16].

Simply put, by reducing the activity of GDH, SIRT4 elicits the inhibition of glutamine anaplerosis and the attendant halt of cell proliferation that provides opportunity for DNA damage repair. Therefore, SIRT4 may attenuate the tumorigenesis by repressing glutamine metabolism and/or genomic instability [145–147].

#### SIRT5

SIRT5 has been considered as a potential oncogene by suppressing PDH, which may facilitate aerobic glycolysis [148]. In support of this notion, SIRT5 is overexpressed in non-small cell lung cancer [149] and ovarian carcinoma [150]. In accord with other sirtuins, SIRT5 is also emerged as a tumor suppressor in squamous cell carcinoma [151] and endometrial carcinoma [152].

#### SIRT6

SIRT6 is regarded as a tumor suppressor partly due to its pivotal role in cancer metabolism. Studies show SIRT6 represses aerobic glycolysis in cancer cells and SIRT6 deficiency contributes to tumor formation even without any oncogene activation [153], indicating the glucose metabolic reprogramming is not a mere consequence of tumorigenesis but also one of its main drivers. The avid glucose take up in SIRT6-deficient cells is due to the fact that SIRT6 binds and co-represses HIF-1α transcriptional activity which suppresses the expression of several key glycolytic genes [42]. In addition, loss of SIRT6 leads to the increasing glutamine metabolism and ribosomal gene expression which are the later events in the tumorigenic process. Decreased H3K56 deacetylation at the promoter region by SIRT6 might account for that [153]. Recently Zhang et al. show that SIRT6 inhibits hepatic gluconeogenesis via interacting with p53 and promotes glucose homeostasis [154].

As with SIRT1, SIRT6 plays both tumor suppressing and promoting roles. SIRT6 expression is downregulated in head and neck squamous cell carcinoma, colon, pancreatic, liver and non-small cell lung cancers [151, 155–157]. Conversely, increased SIRT6 expression has been reported in human skin squamous cell carcinoma and pancreatic, prostate and breast cancers, which suggests a poor prognosis and chemotherapy resistance [158–161].

#### SIRT7

Although received comparatively less attention than other sirtuins, SIRT7 appears to have several features that are critical for human cancers. Recent studies reported that SIRT7 has tumor promoting activities. In this regard, SIRT7 is found to be an oncogene in hepatocellular carcinoma, gastric cancer and colorectal cancer, and depletion of SIRT7 suppresses tumor growth [162–164]. Strikingly, overexpression of this sirtuin protects tumor cells against genotoxic damage and facilitates cell survival, suggesting the possibility that SIRT7 acts an oncogenic role by enhancing genome integrity in tumor cells [165].

To sum up, the dichotomous roles of sirtuins in cancer largely revolve around their functions in DNA damage response and cancer metabolism. In general, tumor-suppressive sirtuins inhibit metabolic shift to glycolysis and glutaminolysis, which are both distinct metabolic changes in cancer cells. And they are engaged in tumorigenesis partly through inducing aerobic glycolysis or sustaining genome stability in tumor cells.

## Conclusions

Extensive studies over the past few years have pointed out that sirtuins are involved in processes including energy metabolism, genome integrity and carcinogenesis. Remarkably, several sirtuins play a dual role in cancer depending on various tumor types, stages and microenvironment. Thus, deciphering the underlying mechanisms and conditions which enabling their opposing role in cancer may be one of the main challenges and of tremendous therapeutic significance. Also, further work will be needed to dissect whether or how sirtuins connect and coordinate different hallmarks of cancer such as genomic instability, deregulated cell metabolism and aberrant tumor microenvironment.
